# Case report: Movement-related neuroplasticity in a patient after spinal cord injury in response to task-oriented bimanual training

**DOI:** 10.3389/fnhum.2024.1502517

**Published:** 2025-01-14

**Authors:** Di Ma, Roy Rongyue Zeng, Sofina Sy Chan, Yu Pan, Jack Jiaqi Zhang

**Affiliations:** ^1^Department of Rehabilitation Medicine, Beijing Tsinghua Changgung Hospital, School of Clinical Medicine, Tsinghua University, Beijing, China; ^2^Department of Rehabilitation Sciences, The Hong Kong Polytechnic University, Kowloon, Hong Kong SAR, China

**Keywords:** spinal cord injury, upper limb, robot-assisted training, neuroplasticity, event-related desynchronization

## Abstract

**Background:**

Bimanual motor training is an effective neurological rehabilitation strategy. However, its use has rarely been investigated in patients with paralysis caused by spinal cord injury (SCI). Therefore, we conducted a case study to investigate the effects of robot-assisted task-oriented bimanual training (RBMT) on upper limb function, activities of daily living, and movement-related sensorimotor activity in a patient with SCI.

**Methods:**

A patient with bilateral upper limb paresis due to incomplete cervical SCI underwent 20 sessions of RBMT. Functional recovery was measured using clinical scales for upper limb motor function and activities of daily living. Training-induced neuroplasticity was evaluated using event-related desynchronization (ERD) induced by movement of the right hand (the more affected side), recorded on the electroencephalogram (EEG).

**Results:**

RBMT improved the patient’s upper limb motor function and activity independence. At baseline, our EEG paradigm demonstrated an ipsilateral predominance of movement-related ERD responses over the sensorimotor cortex (SMC) in relation to the moving hand. Following the RBMT, the ERD pattern shifted from being predominantly ipsilateral to a contralateral allocation.

**Conclusion:**

The present case study provides preliminary evidence to support the therapeutic use of RBMT to restore upper limb function in patients with incomplete SCI. The recovery of function following SCI might be related to the rebalancing of sensorimotor activation.

## Background

Spinal cord injury (SCI) is defined as any injury to the spinal cord that results in sensorimotor dysfunction and abnormal reflex responses below the injury level, leading to dependency in activities of daily living (ADL; Thomas et al., 2024; Lo et al., 2016). Intensive, repetitive, and task-oriented training has been proven to be effective in neurological rehabilitation (Waddell et al., 2014). Robot-assisted task-oriented training has been used as an adjuvant to other therapies to facilitate motor function in individuals with limb paralysis caused by SCI (Cheung et al., 2017). Additionally, bimanual training is an effective neurorehabilitation treatment for upper limb disability. Administration of bimanual upper limb training in neurorehabilitation may recruit more sensorimotor pathways from both hemispheres to facilitate motor gains, compared to unimanual training. Although bimanual training has been extensively utilized in stroke rehabilitation, its therapeutic use remains unknown in SCI (Cauraugh and Summers, 2005; Chen et al., 2019). While SCI and stroke have different pathologies and etiologies, they may share similarities in injury-related neural reorganization and recovery mechanisms rooted in motor relearning (Dobkin, 2009). Robot-assisted bimanual task-oriented training (RBMT) is an established program to enhance upper limb neurorehabilitation in patients with neurological disorders, such as stroke (Ma et al., 2022). It may also hold significant promise in the upper limb rehabilitation of patients with SCI (Ho et al., 2023).

Although cortical areas remain intact after SCI, activation of sensorimotor cortex (SMC) is decreased after deafferentation according to the lesion that disrupts sensorimotor pathways (Curt et al., 2002). Thus, neuroplasticity, e.g., cortical reorganization, is pivotal in motor recovery after SCI (Jurkiewicz et al., 2007; Lynskey et al., 2008). For example, motor learning of the impaired upper limb leads to the remapping of SMC and possibly the recruitment of new functional pathways, all resulting in improved outcomes (Mohammed and Hollis, 2018). Event-related desynchronization (ERD) is an index of cortical activation obtained by electroencephalogram (EEG) and represents a decrease of specific rhythmic neuronal activity elicited by an event. The use of ERD allows the reliable probing of cortical reorganization in patients with SCI, in which a stronger movement-related ERD has been found to induce better clinical progression in patients with SCI (Simis et al., 2021; Vázquez-Marrufo et al., 2017; López-Larraz et al., 2015). In this study, we hypothesized that RBMT improves upper limb recovery and activity independence in patients with incomplete cervical SCI, parallel to an increase in movement-related ERD over the affected SMC.

## Materials and methods

### Case description

**Figure 1:**
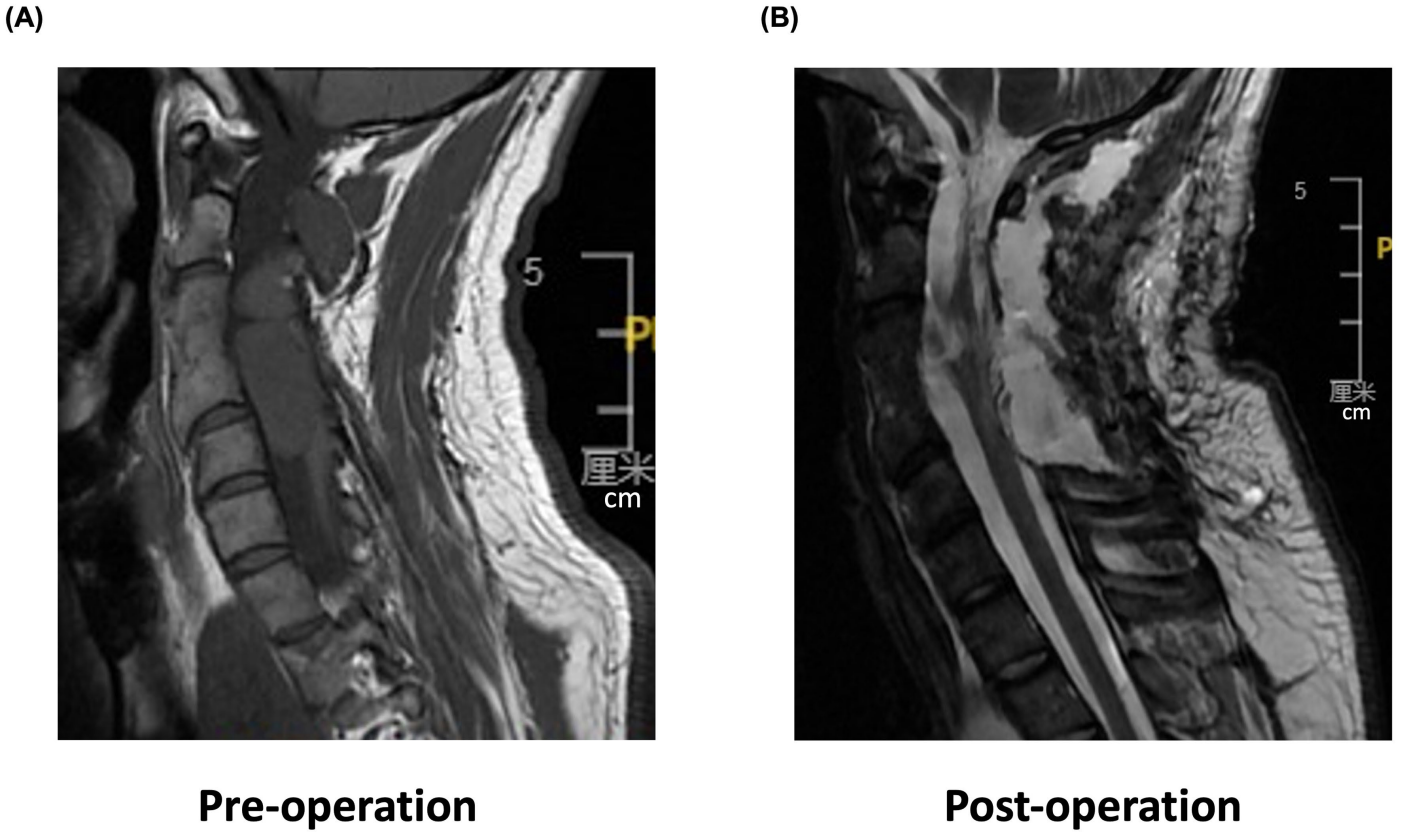
Cervical spine magnetic resonance imaging (MRI). **(A)** Pre-operation sagittal T2-weighted MRI shows a high T2 signal with spinal cord compression in C2–C5 segments. **(B)** Post-operation sagittal MRI reveals morphological changes in C2–C4 segments of the spinal cord with mixed signal intensity and irregular edges.

This study presents a case of incomplete SCI in a 29-year-old man secondary to decompression of a congenital cervical arachnoid cyst with residual neural pathways that are available for motor recovery (Sharma et al., 2023). The patient had experienced neck pain for more than 20 years and reported progressive sensorimotor function impairment upon admission. Magnetic resonance imaging (MRI) and pathological examination revealed a spinal neurenteric cyst, which caused spinal cord compression (Figure 1).

After surgical resection of the cyst in the spinal canal, the patient developed sensorimotor dysfunction, which worsened over time. Further, the patient experienced a loss of pain and temperature sensation below the C3 level on the left side. While left upper limb function improved, the patient failed to elevate his right arm over the shoulder and could not perform fine motor skills, exhibiting severe dependency on ADL. The patient’s grade on the American Spinal Injury Association Impairment Scale (AIS) was D. The left- and right-side sensory and motor levels were at C4 and C5, and C7 and C5, respectively. Upper limb motor examination of the AIS (manual muscle testing) showed that the right upper limb muscle strength (15/25) was lower than that on the left side (20/25), with the right biceps being the lowest (grade 2/5). Similarly, right upper limb function was more affected than on the left side (Action Research Arm Test (ARAT) score: 16/57 vs. 51/57). While the right upper limb showed increased muscle tone (hypertonia, 1+ in MAS) and poor performance on motor coordination tests, the left upper limb with normal muscle tone could normally perform the rapid alternating movement test (one of coordination tests). Somatosensation, including proprioception, vibration, and discriminative touch, was decreased on both sides. In addition, the patient showed an impaired standing balance (static balance level) with a low Berg Balance Scale score (6/56). The patient had engaged in 1-month conventional rehabilitation interventions prior to initiating the RBMT.

### Intervention

Forty-three days after surgery, the patient received 1-h daily RBMT using a wearable robotic system, on 5 consecutive days a week, for a total of 20 days. This training program was designed based on our previous study on upper limb rehabilitation in patients with subacute stroke (Ma et al., 2022). The wearable robotic system comprised a hand exoskeleton, a sensor glove, and a control box (Mirror Hand, HS001, Rehabotics Medical Technology Corporation). The hand exoskeleton, which comprised five individual finger modules that provide three motion modes at a constant speed: five-finger, single-finger, and symmetric movement modes (i.e., mirror therapy), was applied to the most affected hand (i.e., the right hand).

**Figure 2:**
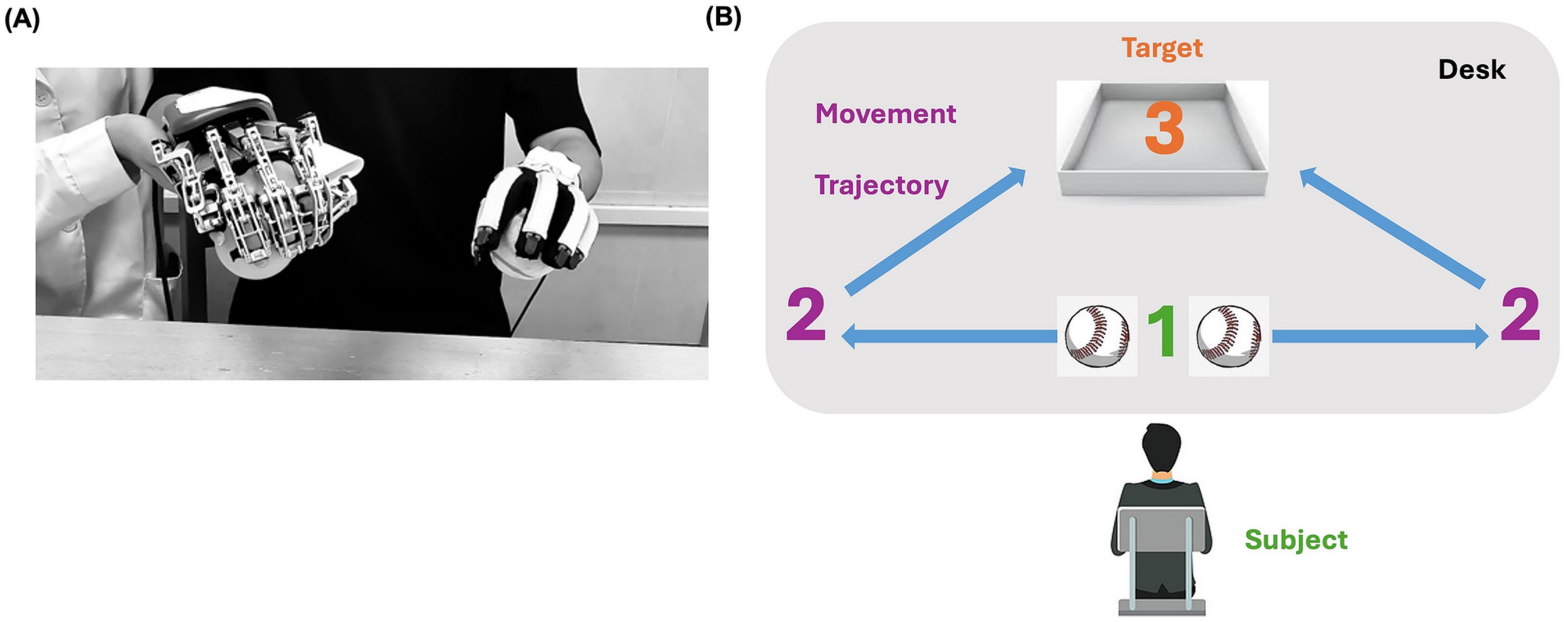
**(A)** The patient wears a hand exoskeleton (right hand) and a sensor glove (left hand) in the starting area. **(B)** The motion trajectory is from 1 to 2 and then to 3.

The training program consisted of three sections executed sequentially. Before each session of RBMT, the patient was instructed to pay attention to the more affected hand and to try to move both hands simultaneously. The procedure was conducted as follows: (1) the patient was asked to move his fingers actively for 5 min while the robot executed five- and single-finger passive movement modes at a speed of 15°/s, assisting the right fingers in performing passive and repetitive extension and flexion movements throughout the full range of motion. (2) A 15-min mirror-guided movement mode was applied to execute hand grasping and opening movements. The exoskeleton mirrored the movement of the sensor glove by receiving position signal from the sensor glove. (3) The patient was asked to perform three hand tasks bilaterally for approximately 30 min. The tasks were selected by an occupational therapist based on the patient’s task performance. Three out of five tasks were selected for this case in each session, including grasping and releasing balls or wooden sticks or conical cylinders and pinching and releasing wooden blocks or pegs. During the tasks, the patient was required to move their hands to predefined areas between the starting and targeting areas (Figure 2). Each task was performed for approximately 10 min, with a 2-min break between tasks. Throughout the RBMT, right-arm elevation was assisted by a suspension device or a therapist.

### Outcome measurements

Clinical assessments of the upper limbs were performed at four time points: (1) before training, (2) after completing 10 sessions, (3) after completing 20 sessions of training, and (4) 2 weeks after training. Assessments included ARAT (0–57, higher scores indicate better performance), the manual muscle test in AIS (0–25, higher scores indicate better muscle function), the modified Ashworth scale (MAS; 0–4, higher scores indicate more severe spasticity), and Spinal Cord Independence Measure (SCIM; 0–100, higher scores indicate greater independence).

EEG measurements were conducted at baseline, after 10 sessions, and after 20 sessions. Movement-related mu rhythm (8–12 Hz) ERD was used as the outcome measure to evaluate neuroplasticity changes after training (Toro et al., 1994). EEG signals were collected using a 64-channel cap (Neuracle, China) sampled at 1,000 Hz. Electrode impedance was maintained below 10 kΩ. We used auditory cues (each lasting 300 ms) with two different tones occurring every 8 s (one to indicate grasping and another for opening) as a ‘go’ signal to trigger hand movements. The patient was instructed to use the right hand (the more affected side) to perform grasping and opening tasks alternatively when hearing the auditory cues. Twenty auditory cued movements were collected in each measurement, with 10 replicates for each task.

EEG data were preprocessed using a bandpass filter from 1 to 40 Hz and re-referenced to the common average. An independent component analysis algorithm was subsequently used to remove the ocular and muscle artifacts. Clean data were analyzed in the time-frequency domain using the *pop_newtimef* function in EEGLAB, with −750 to −50 ms as the baseline to avoid the possible contamination of auditory cues-related neural oscillations, and 300 to 1,000 ms as the task period (Fong et al., 2021). ERD over the SMC was represented by averaging ERD from channels (C3, C1, CP3, and CP1 for the left side and C4, C2, CP4, and CP2 for the right side).

## Results

**Figure 3:**
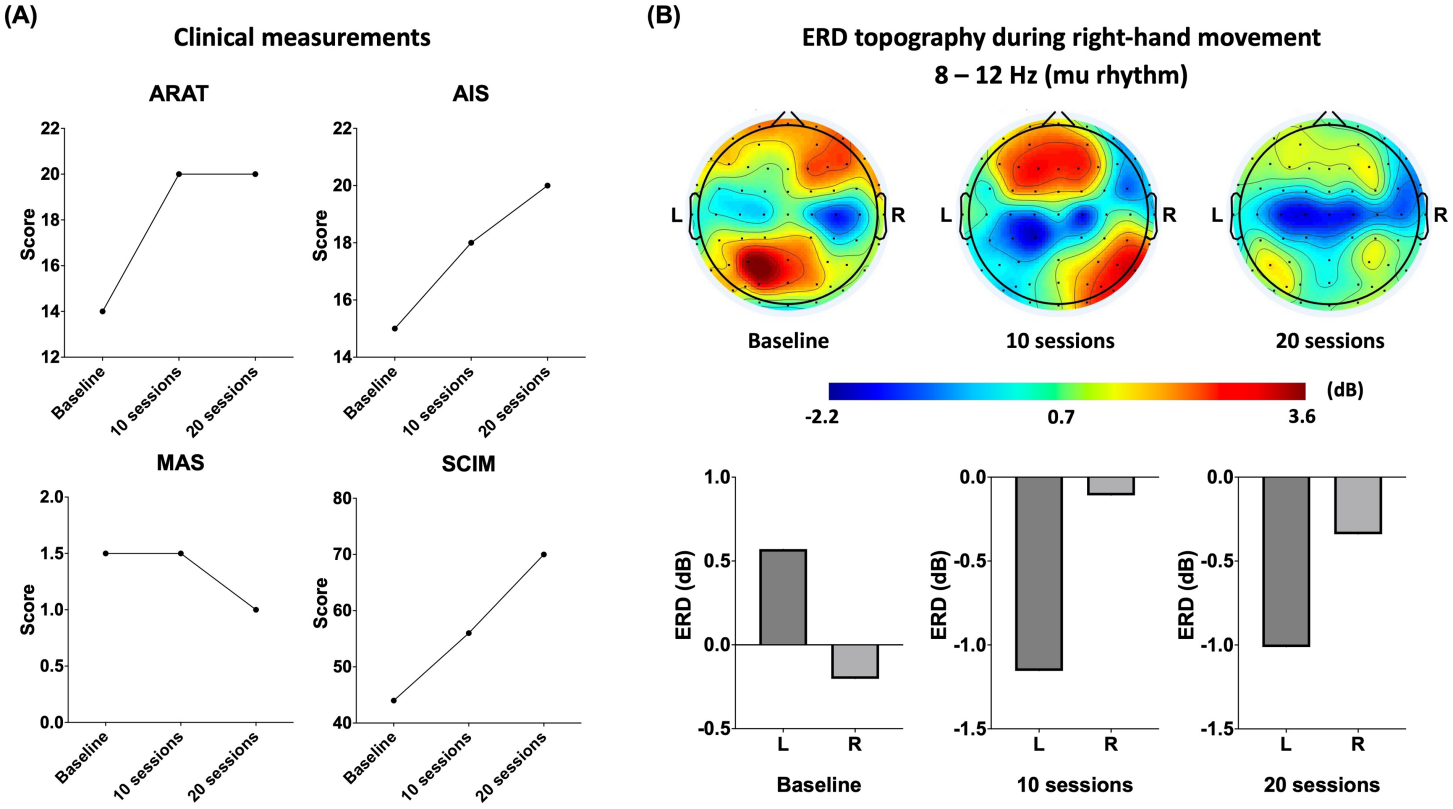
**(A)** Changes in the right upper limb function and activity independence, including the score changes in the Action Research Arm Test (ARAT), the upper limb motor score changes in the American Spinal Injury Association Impairment Scale (AIS), the score changes in the modified Ashworth scale (MAS; 1.5 represents 1+), the score changes in the Spinal Cord Independence Measure (SCIM). **(B)** Changes of mu rhythm during the right-hand movement. On the above are topographic maps on a time window of 300 ms to 1,000 ms, with 0 ms being auditory cues administration. On the bottom are the statistical comparisons of the ERD over the SMC. Bar charts show the mean ERD from channels (C3, C1, CP3, and CP1 for the left SMC and C4, C2, CP4, and CP2 for the right SMC). L: left hemisphere; R: right hemisphere.

We focused on the outcomes from the right upper limb since the left upper limb was mildly impaired and acted as the dominant to drive the hand exoskeleton throughout the training. The RBMT improved the right upper limb motor function over time, as reflected by the improvements in the aforementioned scales, and progressively increased mu-ERD in the contralateral SMC (Figure 3).

After 10 training sessions, the patient’s right upper limb AIS motor score increased from 15/25 to 18/25: elbow flexors (2 to 3), elbow extensors (3 to 4), and finger flexors (3 to 4), while the score of the left side remained the same (20/25). The right upper limb performing ARAT was improved (from 14/57 to 20/57), specifically on gross movement tests (from 0/9 to 3/9) and pinch tests (from 3/18 to 6/18). The left upper limb also performed ARAT better (from 51/57 to 53/57). The total score of SCIM was improved from 44 to 56, specifically on self-care (from 7/20 to 9/20) and mobility (from 6/40 to 16/40). However, same as baseline, spasticity remained 1+ in MAS after 10 sessions. As seen in our video recording, the patient was able to use his thumb and ring finger in pinching up small objects after the 10-session training program (shown in the Supplementary material).

After 20 training sessions, the patient’s AIS right upper limb motor score continuously increased to 20/25, including the elbow flexors (3 to 4) and wrist extensors (3 to 4). Although there was no change in the ARAT at this time, decreased spasticity from 1+ to 1 was observed in the right elbow flexors. In contrast, the left upper limb reached the ceiling of AIS and ARAT after 20 sessions from 20/25 to 25/25 and from 53/57 to 57/57, respectively. The total SCIM score increased further from 56 to 70, specifically on self-care (from 9/20 to 16/ 20), respiration and sphincter management (from 31/40 to 31/40), and mobility (from 16/40 to 22/40). All results persisted at 2 weeks follow-up, during which the SCIM increased continuously in self-care (from 16/20 to 18/20) and mobility (from 22/40 to 25/40).

Movement-related ERD (right hand grasping/opening in this case) over the bilateral SMC changed from a ipsilateral-predominant pattern to a relatively contralateral-predominant pattern, showing the engagement of contralateral SMC during voluntary movement execution.

## Discussion

This case study presents the beneficial effects of RBMT in an adult following surgical treatment for incomplete cervical SCI. The patient showed good adherence and tolerance to the RBMT program, and no adverse events developed. Overall, the patient showed improvements in upper limb function, activity independence, and an increase of sensorimotor activity over the more affected SMC.

Clinical improvements were represented as continually increasing AIS motor examination scores during training, and increased pinch and gross movement scores in the ARAT. These improvements remained stable for at least 14 days after training. However, the improvement of ARAT was limited due to the patient’s persistent right shoulder dysfunction. Most ARAT tasks require lifting small objects. By that, the patient was unable to fully complete the ARAT tasks as his proximal upper limb function did not markedly improve. Nevertheless, our video recordings (shown in the Supplementary material) showed that the patient’s distal upper limb function was significantly enhanced following RBMT.

Baseline EEG revealed a weak ERD in the contralateral SMC during right-hand movements, meaning low activation of the left SMC (Kaiser et al., 2012). After 10 training sessions, the magnitude of the ERD increased in the contralateral SMC, indicating this area may have engaged in motor learning to restore its function (Dietz, 2012). Of note, RBMT involves the mechanisms underlying ‘mirror therapy’, including visual feedback and cross education, as the patient was asked to focus on the more affected hand (Carson and Morley, 2023). This aligns with the extant literature that demonstrated that combining hand exoskeleton devices with mirror therapy yields superior outcomes in enhancing distal upper-extremity function (Li et al., 2023). After completing all 20 sessions, the magnitude of ERD in the left SMC increased continuously, and was higher than that on another side, indicating that the patient would probably achieve greater improvement in the future (Ray et al., 2020). Outcome measures showed ERD was parallelly improved with the clinical assessments. A longitudinal study that stated ERD in mu rhythm was correlated with clinical improvement further supported a potential correlation relationship between them in this case (López-Larraz et al., 2015).

The findings of this study should be interpreted with caution. First, we assessed only one participant. The effects of RBMT may differ for other types of SCI that present with different recovery trajectories, such as complete SCI and pediatric SCI. Second, the selected tasks in RBMT are not centered on ADL. Future studies should carefully design these tasks to be more functional, such as holding a large light-weight ball, which therefore may lead to generalization of the rehabilitation outcome into functional performance. Third, this RBMT is designed for hemiplegia initially; this strategy may not be useful for patients with complete SCI who lack hand movement bilaterally. Finally, kinematic measures were not applied in this study. Future studies should apply rigorous methodology to examine the current results and other advanced outcome measures to unveil the mechanisms underlying functional improvements on the SCI (Nakayashiki et al., 2014).

## Conclusion

RBMT is a promising upper extremity recovery strategy for patients with SCI. The observation that motor function improved in parallel with the changes in ERD pattern over time indicates a possible relationship between mu-ERD and upper limb function.

## Data Availability

The original contributions presented in the study are included in the article/Supplementary material, further inquiries can be directed to the corresponding authors.
